# Differential Expression of Long Noncoding RNAs Reveals a Potential Biomarker for Intractable Pemphigus

**DOI:** 10.1155/2021/5594659

**Published:** 2021-09-03

**Authors:** Peng Qu, Zixuan Huang, Haiqin Zhu, Jie Zheng, Meng Pan

**Affiliations:** ^1^Department of Dermatology, Rui Jin Hospital, Shanghai Jiao Tong University School of Medicine, Shanghai, China; ^2^Shandong Provincial Hospital for Skin Diseases and Shandong Provincial Institute of Dermatology and Venereology, Shandong First Medical University & Shandong Academy of Medical Sciences, Jinan, Shandong, China

## Abstract

**Background:**

Long noncoding RNAs (lncRNAs) are involved in autoimmune diseases. However, the role of lncRNAs in pemphigus remains elusive.

**Objective:**

The study is aimed at investigating the expression profile in pemphigus patients to identify a circulating lncRNA as a novel biomarker for pemphigus.

**Method:**

A global lncRNA expression profile in peripheral blood mononuclear cells (PBMCs) was measured by lncRNA microarray. Differentially expressed lncRNAs were validated by quantitative reverse transcriptase-PCR (qRT-PCR). The functional and biological processes of the differentially expressed lncRNAs were analyzed by bioinformatics.

**Results:**

lncRNA ENST00000585297 in the PBMCs of pemphigus patients was highly expressed compared with those of HCs and BP patients. The area under the receiver operating characteristic (ROC) curve was 0.846 (95%confidence interval (CI) = 0.7526 to 0.9397). Intriguingly, we found that the expression of ENST00000585297 was upregulated in pemphigus patients whose symptoms could not be managed within four weeks compared to other patients whose symptoms could be managed in four weeks or less (*P* < 0.05). In addition, ENST00000585297 expression in pemphigus patients was positively correlated with the dosage of prednisone needed to manage the disorder (*r* = 0.4905, *P* = 0.0094). A competing endogenous RNA (ceRNA) regulatory network was constructed based on the ceRNA theory. Further verification demonstrated that silencing of ENST00000585297 increased the expression of miR-584-3p.

**Conclusions:**

Our study revealed for the first time the expression profile of lncRNAs in pemphigus patients. In addition, our study identified ENST00000585297 as a biomarker and indicator for the intractable course of pemphigus.

## 1. Introduction

Pemphigus is a chronic, recurrent, potentially life-threatening autoimmune bullous disease characterized by flaccid blisters and erosions of mucous membranes and/or the skin. If untreated, it is almost always fatal. The blood of patients with pemphigus contains autoantibodies that mainly target desmoglein (Dsg), a critical glycoprotein found predominantly in the skin epidermis [1, 2]. A series of prior studies and still-accumulating data showed that these antibodies are directly pathogenic; that is, these antibodies that target Dsg can cause a loss of keratinocyte cell adhesion, which results in blistering [3]. Pemphigus is generally managed with oral corticosteroids. However, we observed that sometimes even in patients with similar severity and antibody titers who are using the same recommended corticosteroid dosage, some individuals get better, while others do not. In addition, the potential relapse risk is also a serious problem that clinicians must face. Therefore, a reliable, specific, and sensitive biomarker for evaluation of intractable pemphigus is urgently needed. However, there is no widely accepted biomarker for pemphigus except anti-Dsg antibodies.

Epigenetics refers to stable and heritable changes in gene expression without changing in the underlying DNA sequence. Epigenetic mechanisms include DNA methylation, posttranslational modifications to histone proteins, and regulation of gene expression by noncoding RNA [4]. In addition, increasing evidence has shown that epigenetic changes are involved in the pathogenesis of several autoimmune diseases [5, 6]. To date, dysregulated histone modification, DNA methylation, and miRNA deregulation have been found to play important roles in pemphigus [7, 8]. However, the role of long noncoding RNAs (lncRNAs) in pemphigus remains elusive. lncRNAs are generally defined as a novel group of noncoding RNAs greater than 200 nucleotides with a role in the regulation of several cellular processes such as proliferation, migration, differentiation, and apoptosis [9]. lncRNAs are remarkably versatile molecules that can regulate the expression of protein-coding genes through interactions with other RNAs, DNA, or proteins [10]. The function of lncRNAs is closely related to subcellular localization. In the nucleus, lncRNAs regulate gene expression at the epigenetic and transcriptional levels, and in the cytoplasm, they regulate gene expression at the posttranscriptional and translational levels [11]. Currently, increasing evidence has shown that lncRNAs, which have been primarily studied in the context of genomic imprinting and cell differentiation, are now emerging as key regulators of diverse biological processes [12], especially in immune cells, and molecular mechanisms of autoimmunity [13]. Recent studies have suggested that lncRNAs might be associated with numerous autoimmune diseases [14–17], suggesting that lncRNAs may open a new avenue for studying pemphigus. More recently, lncRNA variations have been identified in endemic pemphigus foliaceus [18]. However, the potential roles of lncRNAs in pemphigus remain unknown.

In the present study, a number of differentially expressed lncRNAs in peripheral blood mononuclear cells (PBMCs) of pemphigus patients were identified using a microarray experiment as an initial approach to investigate the potential functions of lncRNAs in pemphigus. To the best of our knowledge, this is the first study to evaluate the expression profiles of lncRNAs in PBMCs from Chinese pemphigus patients and healthy controls (HCs) in a transcriptome-wide study. In addition, ENST00000585297 was identified as a novel lncRNA biomarker for pemphigus and an indicator for the intractable course of pemphigus.

## 2. Materials and Methods

### 2.1. Subjects and Study Design

Patients with pemphigus were diagnosed based on clinical manifestations and histology criteria (epidermal acantholysis), and details of the patients are shown in Table 1. The study was approved by the Shanghai Jiao Tong University School of Medicine Research Ethics Committee. Written informed consent was obtained from all subjects before the study. 56 pemphigus patients, 35 HCs, and 16 bullous pemphigoid (BP) patients were recruited from the hospital during the same period. The HCs were recruited from the Physical Examination Center of the same hospital. All participants were of the Han Chinese ethnic group and from all cities and provinces of China. The disease activity and severity were quantified according to the Pemphigus Disease Area Index (PDAI) [19].

### 2.2. PBMC Isolation and RNA Isolation

PBMCs were isolated from pemphigus patients, HCs, and BP patients by density separation gradient using Lymphoprep (Stemcell Technologies, Vancouver, Canada) within 4 h of collecting the whole blood (4 ml). Total RNA was extracted from PBMC samples of patients and HCs after lysis with the TRIzol Reagent (Invitrogen Life Technologies, Grand Island, NY, USA) following the manufacturer's instructions. And a NanoDrop spectrophotometer (ND-2000, NanoDrop Technologies, Wilmington, DE) was used for RNA quality control. Only samples with an eligible quantity and quality were used for Arraystar LncRNA Microarrays and quantitative reverse-transcription PCR (qRT-PCR).

### 2.3. Microarray Analysis

Arraystar Human LncRNA Microarray V4.0 was used to screen differentially expressed lncRNAs and mRNAs in PBMCs of pemphigus patients. In this study, PBMC samples from two pemphigus vulgaris (PV) and two pemphigus foliaceus (PF) patients and four age- and sex-matched HCs were selected for microarray analysis. These four newly diagnosed patients had not received immunosuppressive treatment before. Sample labeling, microarray hybridization, and washing were performed based on the manufacturer's standard protocols. After washing, the arrays were scanned by the Agilent DNA Microarray Scanner (part number G2505C).

### 2.4. lncRNA Classification

A lncRNA can be placed into one or more of five broad categories: sense, antisense, bidirectional, intronic, and intergenic [20]. Sense or antisense lncRNAs are RNA molecules that are transcribed from the same or opposite strand, respectively, and overlap with one or more exons of another transcript. Bidirectional lncRNAs share the same promoter with a protein-coding gene on the opposite chain, but the transcription direction of the lncRNA is opposite to that of the protein-coding gene. Intronic lncRNAs are located completely in the intron region of another transcript. Intergenic lncRNAs are transcribed from sequences located between two protein-coding genes.

### 2.5. qRT-PCR Validation of Candidate lncRNAs

Using fold-change filtering (fold change > 2) and padj < 0.05, nine top upregulated lncRNAs were selected and validated by qRT-PCR. Total RNA was reverse transcribed into complementary DNA (cDNA) with the Thermo Scientific RevertAid First Strand cDNA Synthesis Kit (Thermo Fisher Scientific, Waltham, MA), and qRT-PCR was performed using PowerUp™ SYBR™ Green Master Mix (Thermo Fisher Scientific, Waltham, MA) according to the manufacturer's protocol. Amplification was performed in an ABI Vii7 Real-time PCR System (Applied Biosystems, Foster City, CA, USA). The relative expression levels of ENST00000585297 in PBMCs were calculated using the 2^−ΔΔ*C*_*t*_^ method and normalized to *β*-actin expression. For qRT-PCR, the gene-specific primers are shown in Table 2.

### 2.6. RNA Fluorescence in Situ Hybridization (FISH)

The FISH assay was performed to detect and localize ENST00000585297 in PBMCs of PV patients. The probes of ENST00000585297 were synthesized by the RiboBio Company (China) and labeled with Cy3 fluorescent dye. The Ribo™ FISH Kit (RiboBio Company, Guangzhou, China) was used to carry out RNA FISH assay according to the procedure provided by the manufacturer.

### 2.7. Nuclear-Cytoplasmic Fractionation and qRT-PCR

The isolation of cytoplasmic and nuclear RNAs in PBMCs was performed using the PARIS™ kit (Thermo Fisher Scientific) following the manufacturer's instructions. After purification and DNase I treatment, the subcellular fractions were subjected to qRT-PCR. U6 (nucleus control) and GAPDH (cytoplasm control) were used for normalization. The primers used for qRT-PCR are listed in Table 2.

### 2.8. Bioinformatics Analysis

The competing endogenous RNA (ceRNA) regulatory network was constructed to predict the potential molecular mechanism in pemphigus based on the ceRNA hypothesis. We used the prediction databases miRanda and TargetScan to locate the miRNA targets of ENST00000585297. A ceRNA network was then established and visualized using Cytoscape (http://www.cytoscape.org). Pathway analysis and Gene Ontology (GO) analysis for differentially expressed mRNAs were used to explore the potential role of ENST00000585297.

### 2.9. Cell Culture and Transient Transfection

PBMCs were cultured in Roswell Park Memorial Institute (RPMI) 1640 medium (Gibco, Carlsbad, CA, USA) with 10% fetal bovine serum at 37°C with 5% CO_2_ for 24 h before transfection. ENST00000585297-siRNA and negative control (NC-siRNA) were designed and synthesized by GenePharma (Shanghai, China). Transient transfections were performed using Entranster™-R4000 (Engreen Biosystem, Beijing, China) to transfect the ENST00000585297-siRNA or NC-siRNA into PBMCs of pemphigus patients according to the manufacturer's instructions. The inhibition efficiency of specific siRNAs was detected by qRT-PCR. Cells were collected at 24 h after transfection for subsequent experiments.

### 2.10. Statistical Analysis

Statistical analysis was performed by GraphPad (version 8.0). The experimental data are shown as the mean ± SD. Statistical evaluation was determined using the Mann–Whitney test or the one-way analysis of variance (ANOVA), followed by the Bonferroni posttest. The Spearman test was used for correlation studies. A value of *P* < 0.05 was considered significant. The discriminative value in comparing pemphigus patients and HCs was evaluated with a receiver operating characteristic (ROC) curve.

## 3. Results

### 3.1. Expression Profile of lncRNAs in Pemphigus

**Figure 1:**
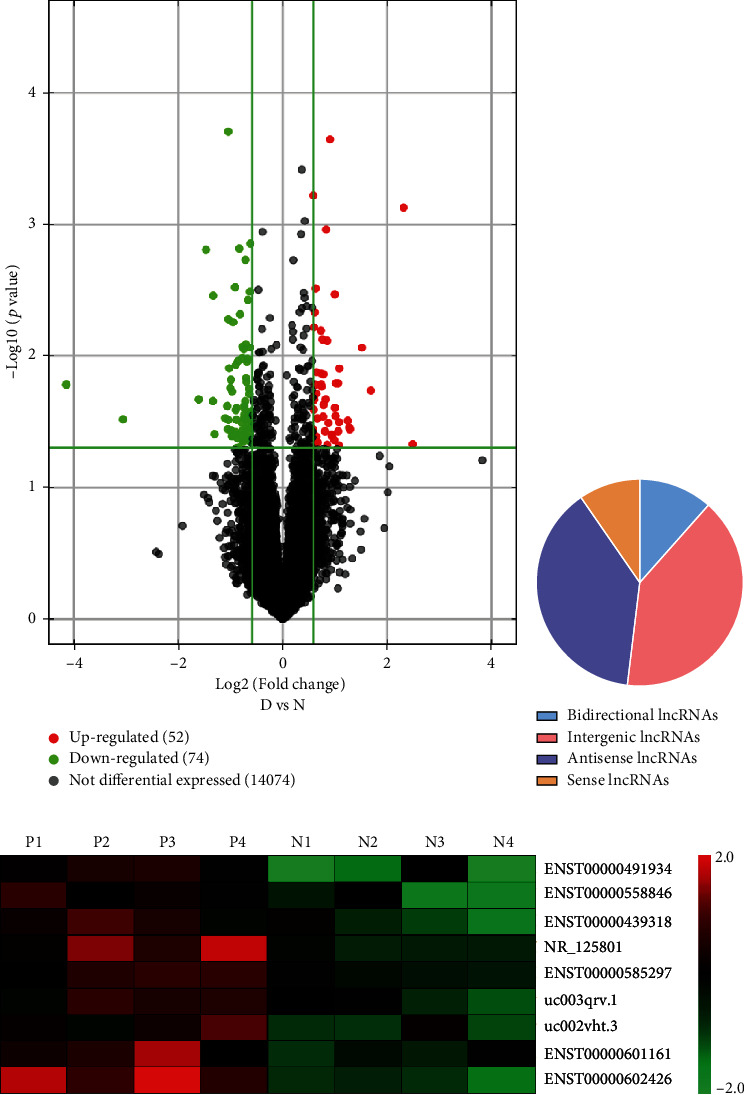
Microarray data on differential lncRNA expression profiles in PBMCs from pemphigus patients and normal controls. (a) Volcano plot of differentially expressed lncRNAs. The green spots indicate significantly downregulated lncRNAs, and the red spots indicate significantly upregulated lncRNAs. (b) The percentage of significantly differentially expressed lncRNAs arising from different genomic regions. (c) Hierarchical clustering of the nine differentially expressed lncRNAs. Red represents relatively overexpressed lncRNAs, and green represents relatively underexpressed lncRNAs.

The lncRNA expression microarray was performed to determine the expression profile of lncRNAs in PBMC samples from four pemphigus patients and four HCs. The scatter plot with differentially expressed lncRNAs (*P* < 0.05, and fold change > 1.5) from microarray analysis is illustrated in Figure 1(a). A total of 126 lncRNAs (52 upregulated and 74 downregulated lncRNAs) were differentially expressed in the pemphigus patient group compared with those in the HC group. Of the 52 upregulated lncRNAs identified, we observed 6 (11.5%) bidirectional, 21 (40.4%) intergenic, 20 (38.5%) antisense, and 5 (9.6%) sense lncRNAs (shown in Figure 1(b)).

### 3.2. ENST00000585297 Might Be a Novel Biomarker in Pemphigus Patients

Among the upregulated lncRNAs in the microarray analysis, the nine top upregulated lncRNAs were selected as candidate lncRNAs for qRT-PCR validation in another sample group that included nine pemphigus patients and nine HCs based on the fold change, *P* value, and primer specificities. Thus, these nine lncRNAs were further studied in the following experiments: ENST00000491934, ENST00000558846, ENST00000439318, ENST00000585297, NR_125801, uc002vht.3, uc003qrv.1, ENST00000601161, and ENST00000602426. These nine differentially expressed candidate lncRNAs were visualized using a heat map (shown in Figure 1(c)).

**Figure 2:**
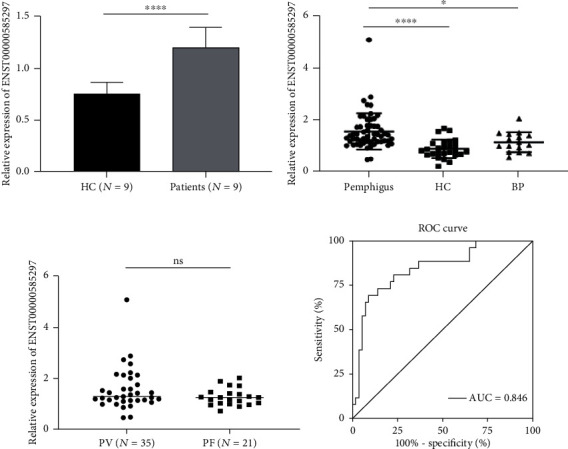
Validation of ENST00000585297 as a pemphigus biomarker. (a) qRT-PCR was conducted on RNA samples from nine pemphigus patients and nine HCs. The expression trends of ENST00000585297 were consistent with the array results. (b) Expression of ENST00000585297 was upregulated in pemphigus patients (*n* = 47) compared with healthy controls (*n* = 26) (*P* < 0.0001) and BP patients (*n* = 16) (*P* < 0.05). (c) No significant difference of ENST00000585297 was seen between the PV group (*n* = 35) and the PF group (*n* = 21) (*P* > 0.05). (d) The ROC analysis for detection of pemphigus patients from healthy controls using circulating ENST00000585297 (^∗^*P* < 0.05 and ^∗∗∗∗^*P* < 0.0001; ns: *P* > 0.05).

As shown in Figure 2(a), the expression level of ENST00000585297 was significantly increased in pemphigus patients (*P* < 0.0001), whereas the other eight lncRNAs had no significant differences in their expression levels between nine pemphigus patients and nine HCs (shown in Figure S1), as determined using qRT-PCR.

### 3.3. Validation of ENST00000585297 as a Pemphigus Biomarker

To further explore whether ENST00000585297 could distinguish between pemphigus patients and HCs or BP patients, we tested it in a cohort of 47 pemphigus patients, 26 HCs, and 16 BP patients using qRT-PCR. The results showed that the expression level of ENST00000585297 in pemphigus patients was higher than that in HCs (*P* < 0.0001) and BP patients (*P* < 0.05; shown in Figure 2(b)). There was no significant difference of expression of ENST00000585297 in PV and PF patients (*P* > 0.05; shown in Figure 2(c)). To evaluate the potential utility of ENST00000585297 as a biomarker for pemphigus, ROC analysis was conducted. When distinguishing pemphigus patients from HCs, the area under the ROC curve (AUC) of the expression level of ENST00000585297 was 0.846 (95% CI: 0.7526-0.9397) (shown in Figure 2(d)), suggesting that ENST00000585297 may be a promising biomarker for pemphigus.

### 3.4. Associations between the Expression Level of ENST00000585297 and Clinical Features

**Figure 3:**
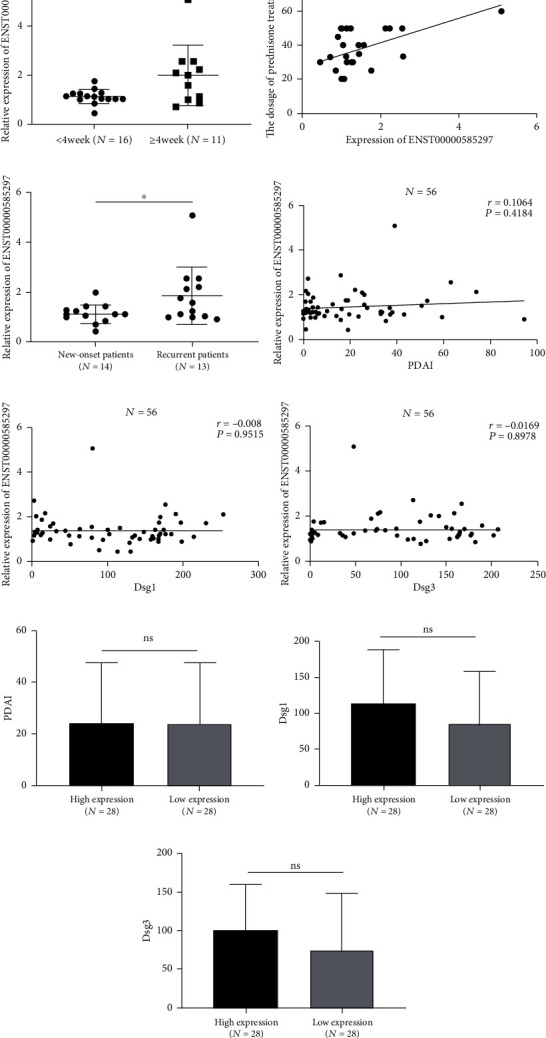
Associations between the expression level of ENST00000585297 and clinical features. (a) Expression of ENST00000585297 was upregulated in pemphigus patients whose symptoms could not be managed within four weeks (*n* = 16) compared with other patients whose symptoms could be managed in four weeks or less (*n* = 11) (*P* < 0.05). (b) ENST00000585297 expression in pemphigus patients (*n* = 27) was positively correlated with dosage of prednisone treatment (*r* = 0.4905, *P* = 0.0094). (c) The expression of ENST00000585297 of the recurrent pemphigus patients were higher than those in the new-onset patients (*P* < 0.05). (d–f) No significant correlation was observed between ENST00000585297 and PDAI or antibody titers in pemphigus patients (*P* > 0.05, *n* = 56). (g–i) No significant difference of PDAI, Dsg1, or Dsg3 was seen between the two groups (*P* > 0.05). (^∗^*P* < 0.05, ^∗∗^*P* < 0.001, and ^∗∗∗∗^*P* < 0.0001; ns: *P* > 0.05).

To explore whether ENST00000585297 might be a potential biomarker in the clinical estimation of the severity and intractability of pemphigus, data on clinical variables were collected from pemphigus patients and the correlation between these data and ENST00000585297 was tested. Interestingly, the results showed that the expression of ENST00000585297 was upregulated in pemphigus patients whose symptoms could not be managed within four weeks compared to other patients whose symptoms could be managed in four weeks or less (*P* < 0.05, shown in Figure 3(a)). ENST00000585297 expression in PBMCs of pemphigus patients was positively correlated with the dosage of prednisone needed to manage the disorder (*r* = 0.4905, *P* = 0.0094; shown in Figure 3(b)). We further analyzed the expression level of ENST00000585297 in the new-onset and recurrent patients. As shown in Figure 3(c), the expression of ENST00000585297 of the recurrent patients was higher than those of the new-onset patients (*P* < 0.05). However, no obvious correlations were observed between ENST00000585297 and PDAI or antibody titers (*P* > 0.05, shown in Figures 3(d)–3(f)). Based on the results of qRT-PCR, the median expression of ENST00000585297 was used as the demarcation point to divide pemphigus patients into the high expression group and the low expression group. The results showed that there was no significant difference of PDAI, Dsg1, or Dsg3 between the two groups (*P* > 0.05, shown in Figures 3(g)–3(i)).

### 3.5. ENST00000585297 May Be Involved in ceRNA Mechanism in Pemphigus

**Figure 4:**
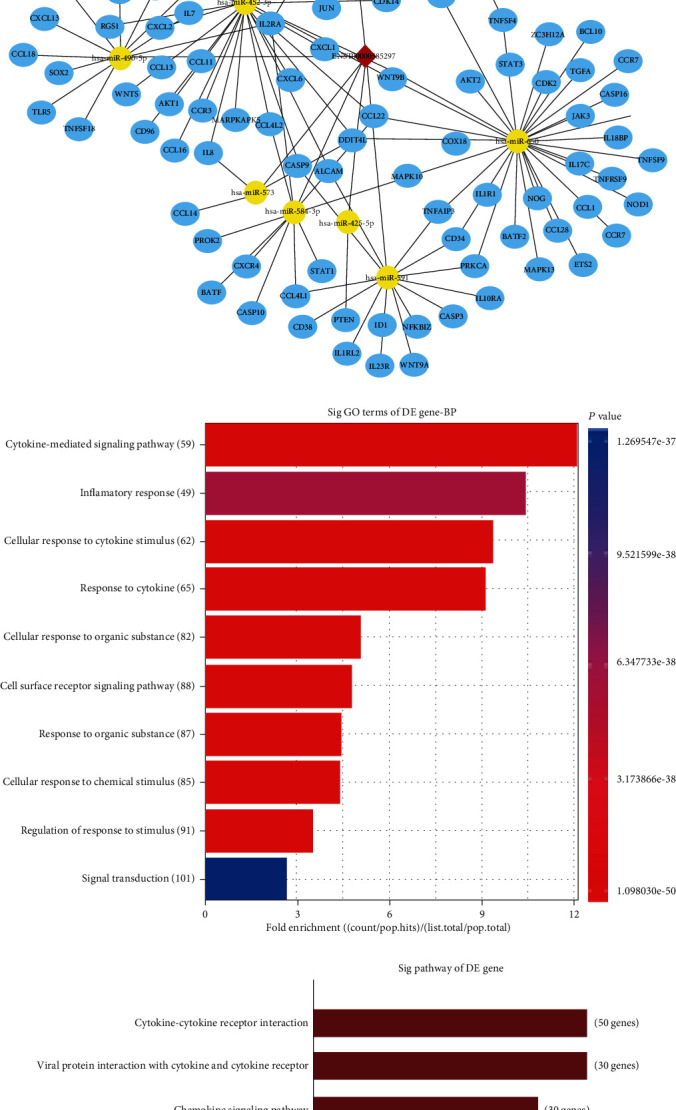
Potential role of ENST00000585297 in pemphigus. (a) RNA FISH assay of ENST00000585297 in pemphigus. (b) qRT-PCR analysis of nuclear and cytoplasmic RNAs showed that ENST00000585297 was preferentially localized within the cytoplasm. (c) The ceRNA network. The blue circle node indicates mRNA; the yellow circle node indicates miRNA; the red diamond indicates lncRNA. (d) GO analysis of predicted mRNA. (e) Pathway analysis of predicted mRNA. (f) ENST00000585297-siRNA efficiently interferes with ENST00000585297 expression in PBMCs (*n* = 3). (g) The relative expression of miR-584-3p between the NC-siRNA and ENST00000585297-siRNA groups (*n* = 5), measured by quantitative reverse-transcription PCR. (^∗∗^*P* < 0.001 and ^∗∗∗∗^*P* < 0.0001).

The subcellular localization of ENST00000585297 was detected by subcellular fractionation assay and RNA FISH. The outcomes showed that most of the ENST00000585297 are located in the cytoplasm, while a small part is located in the nucleus (shown in Figures 4(a) and 4(b)). Mechanistically, the ceRNA pattern is widely reported as the way by which lncRNAs in the cytoplasm regulate gene expressions. Hence, we hypothesized that ENST00000585297 may function as a ceRNA in PBMCs. However, to which miRNAs ENST00000585297 will bind to and with which ceRNA process ENST00000585297 will be involved in remain unclear. Hence, we constructed a ceRNA network based on potential lncRNA/miRNA/mRNA interactions (shown in Figure 4(c)). To explore the underlying mechanism of ENST00000585297 in the pathogenesis of pemphigus, we used these networks to create functional annotations of the predicted target mRNAs by GO and pathway analyses. GO analysis indicated that differentially expressed mRNAs involved in the ceRNA network participated in the cytokine-mediated signaling pathway, response to cytokine, cell surface receptor signaling pathway, cellular response to cytokine stimulus, cellular response to organic substance, response to organic substance, cellular response to chemical stimulus, regulation of response to stimulus, signal transduction, and inflammatory response (shown in Figure 4(d)). Pathway analysis identified 10 pathways that were associated with the upregulated mRNAs. The cytokine-cytokine receptor interaction, viral protein interaction with cytokine and cytokine receptor, chemokine signaling pathway, pathways in cancer, and IL-17 signaling pathway were the top five pathways for the upregulated protein-coding genes (shown in Figure 4(e)). These functions might constitute the foundation of autoimmunity in pemphigus patients.

### 3.6. ENST00000585297 Knockdown Upregulates miR-584-3p Expression in Pemphigus PBMCs

Since the ENST00000585297-microRNA-mRNA network suggested that there may be potential interactions between ENST00000585297 and miRNA, we selected miR-584-3p which was characterized with high comprehensive database scores to study whether knockdown of ENST00000585297 would have an effect on the expression of miR-584-3p. The results showed that compared to the NC-siRNA group, the ENST00000585297-siRNA group showed a significant decrease of expression of ENST00000585297 by 51 ± 2.65% (*P* < 0.0001) (shown in Figure 4(f)). And knockdown of ENST00000585297 in PBMCs significantly increased the level of miR-584-3p, indicating that miR-584-3p may be a potential target of ENST00000585297 (*P* < 0.01, shown in Figure 4(g)).

## 4. Discussion

lncRNAs have been found to play critical roles in the regulation of immune cell differentiation and the activation of innate immunity [21, 22]. However, the exact mechanism of lncRNAs in the pathogenesis of autoimmune diseases is largely underexplored. Some studies have reported that lncRNAs are involved in several autoimmune diseases, such as systemic lupus erythematosus (SLE) and rheumatoid arthritis (RA) [16, 23, 24], but there are no reports on the expression profile of lncRNAs in pemphigus. Therefore, we initiated the study to analyze lncRNA expression profiles in PBMCs of pemphigus patients to investigate the potential functions of lncRNAs in pemphigus.

In our study, we chose nine upregulated lncRNAs and investigated their expression levels in nine pemphigus patients and nine HCs, and only the expression level of ENST00000585297 was significantly upregulated. The conflicting results may result from different sensitivities and specificities of the two methods used in this study, as the veracity of microarrays is generally inferior to that of qRT-PCR. Further qRT-PCR experiments indicated that the expression level of ENST00000585297 was markedly increased in pemphigus patients compared with that in HCs and BP patients, indicating that ENST00000585297 could distinguish pemphigus patients from not only HCs but also BP patients. These results indicated that the expression level of ENST00000585297 in PBMCs may be used as a novel biomarker for pemphigus, and it may play a role in the pathogenesis of pemphigus.

Despite several earlier studies on the role of demographic and clinical factors in the prognosis of pemphigus [25–27], there are no data associated with clinically available biomarkers to predict refractoriness of lesions in pemphigus patients. In our clinical works, we found that in patients with similar severity of illness and antibody titers, patients' response to the same dosage of glucocorticoids may vary between individuals, and some patients tend to need a higher dosage of glucocorticoids to recover. Thus, knowing the intractable course of pemphigus patients before treatment is important. Interestingly, in our study, results indicated that patients with higher levels of ENST00000585297 tend to need more glucocorticoids and time to recover. And there were no significant relations between the expression of ENST00000585297 and disease severity or antibody titers. This may be because ENST00000585297 was unrelated to the severity but was related to the refractoriness of lesions in pemphigus patients. This phenomenon conforms to a previous study which demonstrated that there was no significant association between baseline disease severity and treatment refractoriness [28]. They also found that neither pretreatment nor posttreatment of antidesmoglein 1 and 3 antibody titres was correlated with treatment resistance. In addition, we found that the ENST00000585297 expression level was significantly elevated in recurrent patients. This seems reasonable because recurrent patients are often more difficult to treat and require a higher dosage of glucocorticoids than new-onset patients. These findings suggest that ENST00000585297 may be an indicator for the intractable course of pemphigus.

In 2011, the ceRNA hypothesis was put forward suggesting that some lncRNAs could act as ceRNAs that can crosstalk with RNA transcripts by competing for miRNAs through common binding sites, and these lncRNAs may perform their functions as a molecular sponge of microRNAs [29]. To explore the potential mechanism of ENST00000585297 in pemphigus, we performed a bioinformatics analysis to seek its interactions and targets. The related pathway and downstream genes of ENST00000585297 were predicted using TargetScan, miRanda, KEGG database, and GO analysis. In our study, GO and KEGG analyses both suggested the association of cytokine with pemphigus in the ceRNA network. Numerous studies have confirmed that cytokines are deeply involved in pemphigus pathogenesis. For example, we have reported the presence of both IL-21 and IL-17 in pemphigus lesions, which were secreted by infiltrated CD4+ T follicular helper- (Tfh-) like cells in our previous study [30]. Likewise, several studies have reported increased levels of IL-8, IL-6, and TNF-*α* in pemphigus, which were also in the ceRNA network [31–33]. In addition, we further verified that ENST00000585297 suppression induced a significant upregulation of miR-584-3p. We suggest that ENST00000585297 may be a molecular decoy for miRNA in pemphigus.

In summary, this study provides novel evidence of the differential expression of lncRNAs in pemphigus patients. Although the underlying mechanisms remain elusive, our study represents the first of such efforts to identify lncRNA as a powerful biomarker and indicator of treatment refractoriness in pemphigus. More studies exploring the role of lncRNAs in pemphigus are expected in the future and will be critical for deepening the understanding of and developing an effective therapy for this disorder.

## Figures and Tables

**Table 1:** Clinical characteristics of patients and healthy controls.

Characteristics	Pemphigus patients (*n* = 56)	Healthy controls (*n* = 35)	Bullous pemphigoid patients (*n* = 16)
Age			
Mean ± SD	47.6 ± 17.1	49.2 ± 19.3	59.2 ± 20.3
Male	30	17	9
Female	26	18	7
Pemphigus subtypes			
Pemphigus vulgaris	35	—	—
Pemphigus foliaceus	21	—	—
Disease duration		—	
<1 year	28	—	8
1-5 years	21	—	6
5 years	7	—	2
Disease severity (*n*)			
Mild	22	—	6
Moderate	14	—	4
Severe	20	—	6

**Table 2:** Primer sequences for qRT-PCR.

Gene	Sequence (5′–3′)
ENST00000585297	F: 5′ CTCGGAAGAAGCAGACCCTG 3′
	R: 5′ GGAAAGGAGGCATAGGAAGC 3′
*β*-Actin	F: 5′ GTGGCCGAGGACTTTGATTG 3′
	R: 5′ CCTGTAACAACGCATCTCATATT 3′
ENST00000491934	F: 5′ CCTACTCTTACATTCATGCGTC 3′
	R: 5′ CCTTATTATTTTTGTTATGACCTG 3′
ENST00000558846	F: 5′ GAGACAGACTCGCTTCCACC 3′
	R: 5′ GTGGGGGAGCAGCTTTATGA 3′
ENST00000439318	F: 5′ TGTGCATTCGGAACTCACC 3′
	R: 5′ GGCTGGACATGGCATCTTA 3′
NR_125801	F: 5′ GACGCTAAATGTCCAAAACG 3′
	R: 5′ CCTATGAAGGGCTAGTAACCAA 3′
uc002vht.3	F: 5′ GTGGTGGTATTTGGAGAAAGG 3′
	R: 5′ TCATGGTGCCATCAGGGTT 3′
uc003qrv.1	F: 5′ TGTTACTGGGAGCAGCATTG 3′
	R: 5′ GCAGGCAAAGCAGAAAAGC 3′
ENST00000601161	F: 5′ GAGAAACCCTTTATGAAAGGC 3′
	R: 5′ CAACTGTCTATTTTGTCCTCATAC 3′
ENST00000602426	F: 5′ CCTCTGCTGTAACTATGGTTGTG 3′
	R: 5′ CTGGGTAAAGGGTAAGTGGAAA 3′
hsa-miR-584-3p	GSP: 5′ GGTCAGTTCCAGGCCAA 3′
	R: 5′ GTGCGTGTCGTGGAGTCG 3′
GAPDH	F: 5′ GAAG ATGGTGATGGGATTTC 3′
	R: 5′ GAAGGTGAA GGTCGGAGT 3′
U6	F: 5′ TCGCTTCGGCAGCACATA 3′
	R: 5′ TTTG CGTGTCATCCTTGC 3′

## Data Availability

The qRT-PCR data used to support the findings of this study are included within the supplementary information file(s).

## Additional Points

*Key Message*. Our study identified ENST00000585297 as a biomarker and indicator for the intractable course of pemphigus.

## Supplementary Materials

Supplementary Materials Figure S1: expression of the other eight candidate lncRNAs in PBMCs of pemphigus patients and healthy controls. (A–H) qRT-PCR was conducted for RNA samples from nine pemphigus patients and nine HCs. The expression trends of these lncRNA were inconsistent with the lncRNA microarray profile results. (ns, *P* > 0.05).

## References

[1] Stanley J. R., Koulu L., Klaus-Kovtun V., Steinberg M. S. (1986). A monoclonal antibody to the desmosomal glycoprotein desmoglein I binds the same polypeptide as human autoantibodies in pemphigus foliaceus. *Journal of Immunology*.

[2] Amagai M., Klaus-Kovtun V., Stanley J. R. (1991). Autoantibodies against a novel epithelial cadherin in pemphigus vulgaris, a disease of cell adhesion. *Cell*.

[3] Stanley J. R., Amagai M. (2006). Pemphigus, bullous impetigo, and the staphylococcal scalded-skin syndrome. *The New England Journal of Medicine*.

[4] Flavahan W. A., Gaskell E., Bernstein B. E. (2017). Epigenetic plasticity and the hallmarks of cancer. *Science*.

[5] Pyfrom S., Paneru B., Knox J. J. (2021). The dynamic epigenetic regulation of the inactive X chromosome in healthy human B cells is dysregulated in lupus patients. *Proceedings of the National Academy of Sciences of the United States of America*.

[6] Surace A. E. A., Hedrich C. M. (2019). The role of epigenetics in autoimmune/inflammatory disease. *Frontiers in Immunology*.

[7] Zhao M., Huang W., Zhang Q. (2012). Aberrant epigenetic modifications in peripheral blood mononuclear cells from patients with pemphigus vulgaris. *British Journal of Dermatology*.

[8] Xu M., Liu Q., Li S. (2020). Increased expression of miR-338-3p impairs Treg-mediated immunosuppression in pemphigus vulgaris by targeting RUNX1. *Experimental Dermatology*.

[9] Tan H., Zhang S., Zhang J. (2020). Long non-coding RNAs in gastric cancer: new emerging biological functions and therapeutic implications. *Theranostics*.

[10] Ashrafizaveh S., Ashrafizadeh M., Zarrabi A. (2021). Long non-coding RNAs in the doxorubicin resistance of cancer cells. *Cancer Letters*.

[11] Statello L., Guo C. J., Chen L. L., Huarte M. (2021). Gene regulation by long non-coding RNAs and its biological functions. *Nature Reviews. Molecular Cell Biology*.

[12] Chen B., Deng S., Ge T. (2020). Live cell imaging and proteomic profiling of endogenous NEAT1 lncRNA by CRISPR/Cas9-mediated knock-in. *Protein & Cell*.

[13] Atianand M. K., Caffrey D. R., Fitzgerald K. A. (2017). Immunobiology of long noncoding RNAs. *Annual Review of Immunology*.

[14] Messemaker T. C., Chadli L., Cai G. (2018). Antisense long non-coding RNAs are deregulated in skin tissue of patients with systemic sclerosis. *The Journal of Investigative Dermatology*.

[15] Angiolilli C., Marut W., van der Kroef M., Chouri E., Reedquist K. A., Radstake T. R. D. J. (2018). New insights into the genetics and epigenetics of systemic sclerosis. *Nature Reviews Rheumatology*.

[16] Dong G., Yang Y., Li X. (2020). Granulocytic myeloid-derived suppressor cells contribute to IFN-I signaling activation of B cells and disease progression through the lncRNA NEAT1-BAFF axis in systemic lupus erythematosus. *Biochimica et Biophysica Acta - Molecular Basis of Disease*.

[17] Mo B. Y., Guo X. H., Yang M. R. (2018). Long non-coding RNA GAPLINC promotes tumor-like biologic behaviors of fibroblast-like synoviocytes as microRNA sponging in rheumatoid arthritis patients. *Frontiers in Immunology*.

[18] Lobo-Alves S. C., Augusto D. G., Magalhães W. C. S. (2019). Long noncoding RNA polymorphisms influence susceptibility to endemic pemphigus foliaceus. *The British Journal of Dermatology*.

[19] Rahbar Z., Daneshpazhooh M., Mirshams-Shahshahani M. (2014). Pemphigus disease activity measurements: pemphigus disease area index, autoimmune bullous skin disorder intensity score, and pemphigus vulgaris activity score. *JAMA Dermatology*.

[20] Ashrafizadeh M., Gholami M. H., Mirzaei S. (2021). Dual relationship between long non-coding RNAs and STAT3 signaling in different cancers: new insight to proliferation and metastasis. *Life Sciences*.

[21] Schmitt A. M., Chang H. Y. (2016). Long noncoding RNAs in cancer pathways. *Cancer Cell*.

[22] Flynn R. A., Chang H. Y. (2014). Long noncoding RNAs in cell-fate programming and reprogramming. *Cell Stem Cell*.

[23] Xue Z., Cui C., Liao Z. (2018). Identification of lncRNA Linc00513 containing lupus-associated genetic variants as a novel regulator of interferon signaling pathway. *Frontiers in Immunology*.

[24] Clarke J. (2020). Risk variant in long noncoding RNA linked to SLE. *Nature Reviews Rheumatology*.

[25] Saha M., Bhogal B., Black M. M., Cooper D., Vaughan R. W., Groves R. W. (2014). Prognostic factors in pemphigus vulgaris and pemphigus foliaceus. *British Journal of Dermatology*.

[26] Herbst A., Bystryn J. C. (2000). Patterns of remission in pemphigus vulgaris. *Journal of the American Academy of Dermatology*.

[27] Savin J. A. (2010). Some factors affecting prognosis in pemphigus vulgaris and pemphigoid. *British Journal of Dermatology*.

[28] Kumar S., de D., Handa S. (2017). Identification of factors associated with treatment refractoriness of oral lesions in pemphigus vulgaris. *The British Journal of Dermatology*.

[29] Salmena L., Poliseno L., Tay Y., Kats L., Pandolfi P. P. (2011). A ceRNA hypothesis: the Rosetta stone of a hidden RNA language?. *Cell*.

[30] Yuan H., Zhou S., Liu Z. (2017). Pivotal role of lesional and perilesional T/B lymphocytes in pemphigus pathogenesis. *The Journal of Investigative Dermatology*.

[31] Timóteo R. P., Silva M. V., da Silva D. A. A. (2017). Cytokine and chemokines alterations in the endemic form of pemphigus foliaceus (fogo selvagem). *Frontiers in Immunology*.

[32] Assaf S., Malki L., Mayer T. (2021). ST18 affects cell-cell adhesion in pemphigus vulgaris in a tumour necrosis factor-*α*-dependent fashion. *The British Journal of Dermatology*.

[33] Metwally D., Fawzy M., ElKalioby M. (2020). Assessment of the quality of life, prevalence of depression, and the level of interleukin 6 in patients with pemphigus vulgaris. *Acta Dermatovenerologica Croatica*.

