# Supplementary figures and images for: Differential Expression of Long Noncoding RNAs Reveals a Potential Biomarker for Intractable Pemphigus
Source: Dis Markers. 2021 Sep 3;2021:5594659. doi: 10.1155/2021/5594659 (PMC8440090; doi:10.1155/2021/5594659)

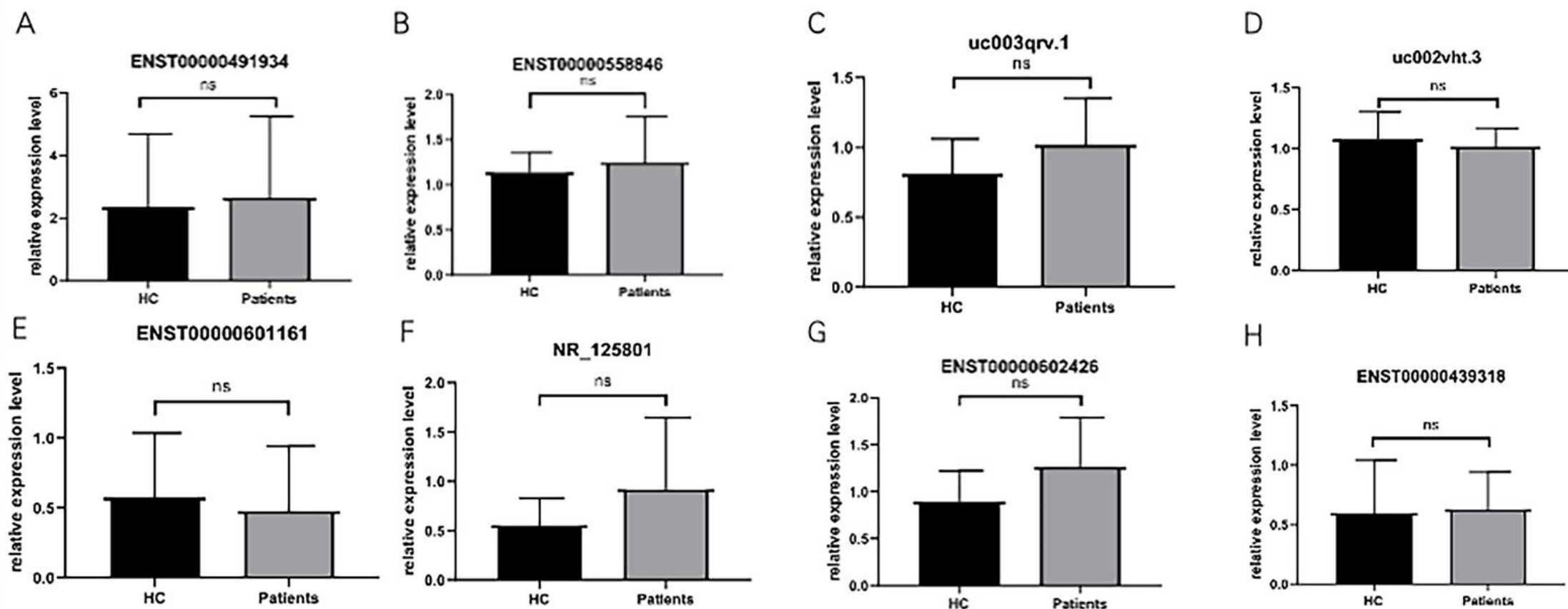

Supplement: Supplementary Materials — Figure S1: expression of the other eight candidate lncRNAs in PBMCs of pemphigus patients and healthy controls. (A–H) qRT-PCR was conducted for RNA samples from nine pemphigus patients and nine HCs. The expression trends of these lncRNA were inconsistent with the lncRNA microarray profile results. (ns, P > 0.05). [file 5594659.f1.pdf]
